# Electrochemical Impedance and Polarization Corrosion Studies of Tantalum Surface Modified by DC Plasma Electrolytic Oxidation

**DOI:** 10.3390/ma11040545

**Published:** 2018-04-03

**Authors:** Maciej Sowa, Wojciech Simka

**Affiliations:** Faculty of Chemistry, Silesian University of Technology, B. Krzywoustego Street 6, 44-100 Gliwice, Poland

**Keywords:** tantalum, anodization, plasma electrolytic oxidation, biomaterials, corrosion, electrochemical impedance spectroscopy, potentiodynamic polarization

## Abstract

Tantalum has recently become an actively researched biomaterial for the bone reconstruction applications because of its excellent corrosion resistance and successful clinical records. However, a bare Ta surface is not capable of directly bonding to the bone upon implantation and requires some method of bioactivation. In this study, this was realized by direct current (DC) plasma electrolytic oxidation (PEO). Susceptibility to corrosion is a major factor determining the service-life of an implant. Therefore, herein, the corrosion resistance of the PEO coatings on Ta was investigated in Ringer’s solution. The coatings were formed by galvanostatic anodization up to 200, 300 and 400 V, after which the treatment was conducted potentiostatically until the total process time amounted to 5 min. Three solutions containing Ca(H_2_PO_2_)_2_, Ca(HCOO)_2_ and Mg(CH_3_COO)_2_ were used in the treatment. For the corrosion characterization, electrochemical impedance spectroscopy and potentiodynamic polarization techniques were chosen. The coatings showed the best corrosion resistance at voltages low enough so that the intensive sparking was absent, which resulted in the formation of thin films. The impedance data were fitted to the equivalent electrical circuits with two time constants, namely *R*(*Q*[*R*(*QR*)]) and *R*(*Q*[*R*(*Q*[*RW*])]). The inclusion of *W* in the circuit helped to fit the low-frequency part of the samples PEO-ed at 400 V, hinting at the important role of diffusion in the corrosion resistance of the PEO coatings described in the research.

## 1. Introduction

In recent years, tantalum has received a considerable amount of attention regarding its application in the biomedical sector. The metal was known to exhibit good biocompatibility since the 1940s [1,2,3]. However, its high cost and density explain why it has not been researched as vigorously as titanium and its alloys. These disadvantages can be amended by coating Ta on top of another material, like carbon [4,5,6,7] or titanium [8,9]. Both Ti and Ta owe their excellent biological performance to the surface properties of the naturally occurring passive oxide films, which are less than 10 nm in thickness [10]. Furthermore, tantalum is characterized by the better corrosion resistance in simulated physiological media, compared to titanium [8,11].

Metallic biomaterials are mostly utilized in bone reconstruction medical devices, such as joint replacement prostheses [12]. Modern materials of this class are usually required not only to remain bio-inert to the implanted organism but also exhibit a specific behavior towards the tissue environment. A specific action of the implant’s surface directed to mobilize the organism to bond the material directly to the bone via hydroxyapatite synthesized by the osteoblasts without fibrous tissue formation is usually described as bioactivity [13]. Such a response is usually encountered for the bioceramics and bioglasses, especially those rich in Ca and P compounds. Therefore, a large array of methods (e.g., like plasma spraying, magnetron sputtering, electrophoretic deposition or sol-gel) for preparation of bioactive ceramic coatings on the metallic implants has been introduced [14].

Among these methods one can name plasma electrolytic oxidation (PEO); also known as micro-arc oxidation (MAO) or micro-plasma oxidation (MPO) [15,16]. The process can be described as a variant of anodization, whereby a metallic anode (e.g., Mg, Ti, Nb, Zr, Al and their alloys [17,18,19,20]) is anodically polarized beyond the dielectric breakdown voltage, which results in the formation of plasma on the treated surface. The existence of the plasma provides a means of producing oxide layers of considerable thickness (up to a few hundred µm) and porosity (external and internal), which increase the surface area available for bone ingrowth. Local heating of the oxide promotes the formation of the phases characteristic for high-temperature processes. In addition, the combined action of the strong electric field and rapid growth of the coating makes it possible to incorporate electrolyte components inside the oxide. By carefully tuning the process parameters it is possible to control the surface properties of the resulting PEO coatings on tantalum [21,22].

The corrosion resistance of the biomaterials used for load-bearing applications is an essential parameter determining their long-term stability. Not only does it encompass the mechanical strength deterioration of the material over time but also the effect of the corrosion products on the human organism, as metal ions may be released from the implant in the process. Therefore, it is widely practiced to test the corrosion susceptibility of the newly formed materials in vitro by immersion studies or electrochemical experiments in the suitable simulated body environments, like Ringer’s or Hank’s saline solutions [23].

In this work, the DC PEO coatings on commercially pure Ta, obtained by the procedure detailed in our previous study [21], were subjected to electrochemical corrosion measurements in Ringer’s physiological solution at 37 °C. Electrochemical impedance spectroscopy (EIS) and potentiodynamic polarization (PDP) methods were used. The coatings were formed in three electrolytes containing calcium hypophosphite, calcium formate, and magnesium acetate. The effect of the electrolytic bath composition and the limiting anodization voltage on the corrosion resistance of Ta was assessed.

## 2. Materials and Methods

**Figure 1:**
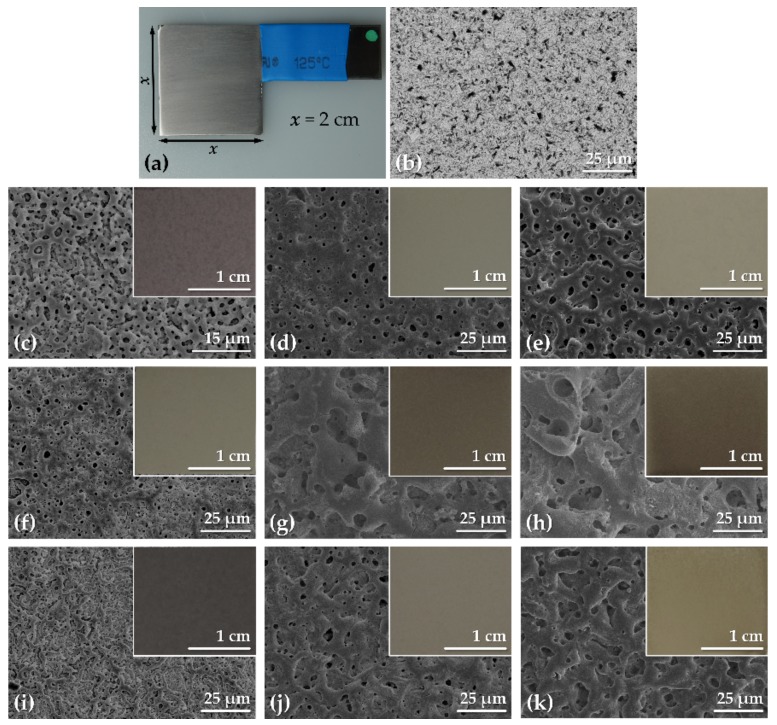
Macroscopic (**a**) and SEM (**b**) images of Ta specimen prior to PEO; SEM images of the Ta surfaces after PEO treatment carried out in the solutions: Ca1P2 (**c**–**e**), Ca5P3 (**f**–**h**) and (CaMg)5P3 (**i**–**k**); and up to the limiting voltages: 200 V (**c**,**f**,**i**), 300 V (**d**,**g**,**j**) and 400 V (**e**,**h**,**k**); insets found in the SEM images are the macroscopic appearances of the respective coatings.

Pure tantalum samples were fabricated from a metal sheet (BIMO Metals, Wroclaw, Poland) and were fashioned into “p”-shaped plates (Figure 1a). The area of the sample that was exposed to the electrolyte during the PEO was 2 cm × 2 cm × 2 cm = 8 cm^2^. The remainder of the metal’s surface area was insulated using a heat-shrink tubing, leaving a small protrusion at the end serving as an electrical contact above the liquid. The complete procedure of the pretreatment and the formation of PEO coatings on Ta was described in our previous study [21]. In short, the samples were mechanically ground using SiC abrasive paper, ultrasonically cleaned (5 min) and then etched in the solution that contained 4 M H_2_SO_4_ and 1 M HF (2 min). The etching was aimed at removing the remainder of the SiC particles after the grinding step. The etched samples were used as a reference throughout the experiments (labeled as Untreated Ta).

The PEO treatment was realized via DC galvanostatic anodization (anodic current density = 150 mA/cm^2^) up to one of the three limiting voltages, *U*_L_ = 200, 300 or 400 V. Three electrolytes were chosen for the study:

1. 0.5 M Ca(H_2_PO_2_)_2_ − molar ratio Ca:P = 1:2; sample label: Ca1P2
2. 0.5 M Ca(H_2_PO_2_)_2_ + 1.15 M Ca(HCOO)_2_ − molar ratio Ca:P = 5:3; sample label: Ca5P3
3. 0.5 M Ca(H_2_PO_2_)_2_ + 1.15 M Mg(HCOO)_2_ − molar ratio (CaMg):P = 5:3; sample label: (CaMg)5P3

After the process voltage reached the *U*_L_, the treatment was conducted under a potentiostatic regime until the total processing time of 5 min was attained. The samples were then washed in running water, followed by thorough rinsing with demineralized water. Thus, cleaned Ta specimens with the PEO coatings were put into a stream of warm air until completely dry.

Macroscopic views of the samples were captured using a digital camera (Sony Alpha ILCE-5000, Tokyo, Japan) after the samples were dried. The investigations of surface morphology were carried out using a scanning electron microscopy (SEM; Hitachi S-3400N, Tokyo, Japan; accelerating voltage, *U*_acc_ = 25 kV; secondary electrons detector), while the cross-sectional SEM images of the PEO coatings were taken using a different device (Phenom ProX, Phenom-World BV, Eindhoven, The Netherlands, *U*_acc_ = 10 kV; back-scattered electrons detector) coupled with an energy-dispersive X-ray spectrometer (EDX) (Phenom ProX, Phenom-World BV, Eindhoven, The Netherlands).

The thickness of the coatings was assessed by taking 3–6 SEM images of the cross-sections and measuring their thickness at 5 thickest and 5 thinnest points. The obtained values for each image were averaged and the corresponding standard deviation (SD) was calculated. The final values for each coating was an average of each image for the given sample and the SD was the largest value among the captured images.

The corrosion resistance of tantalum in the simulated physiological environment, before and after the surface treatment, was estimated by electrochemical methods. Ringer solution (8.6 g/dm^3^ NaCl, 0.3 g/dm^3^ KCl and 0.48 g/dm^3^ CaCl_2_·6H_2_O; Fresenius Kabi, Warsaw, Poland) was chosen as an electrolyte. A 250 mL corrosion cell (Bio-Logic, Seyssinet-Pariset, France), fitted with platinum mesh counter electrode and a salt bridge with the Haber–Ługgin capillary for the reference electrode mounting, was used in the experiments. Saturated calomel electrode (SCE) served as the reference electrode. The sample was exposed to the solution through an opening in the cell with a flat O-ring (exposed surface area = 1 cm^2^). The measurements were made using PARSTAT 4000 potentiostat-galvanostat (Ametek, Princeton Applied Research, Berwyn, PA, USA) and the dedicated Versa Studio software (v. 2.52.3). The following experiments were carried out in a sequential procedure:

- 5 h of open-circuit potential (*E*_OC_) stabilization; *E*_OC_ drift rate was typically below 10 mV/h and did not exceed 16 mV/h for any sample
- Electrochemical impedance spectroscopy performed at *E*_OC_, frequency range 100 kHz–10 mHz and root mean square (RMS) amplitude of 10 mV; the measurement took up to 60 min
- After the potential was stabilized after the EIS experiment, a series of potentiostatic measurements (120 s) were carried out, starting at *E*_OC_ and decreasing down to −30 mV vs. *E*_OC_ with a step of 3 mV; then the potential was allowed to stabilize for 10 min prior to the analogical series of measurements up to +30 mV vs. *E*_OC_; the steady-state value of the measured current at each potential step was used to plot polarization curves near the *E*_OC_ and extract polarization resistance (*R*_p_) in the limit of ±10 mV vs. *E*_OC_
- Immediately after the potentiostatic experiments, the potentiodynamic polarization was conducted up to +2 V vs. SCE; the potential scan rate was 10 mV/min

The third item in the procedure was utilized to avoid excessive charging of the oxide coatings, thus acquiring the current values that were closer to the actual current being spent on faradaic processes. The charging current is getting lower in proportion to its faradaic counterpart as the potential is more distant from *E*_OC_, therefore, the potentiodynamic polarization followed the potentiostatic measurements. 

A minimum of three parallel samples was used for the PEO coatings and the untreated Ta samples. The equivalent electrical circuits (EECs) were devised and fitted to the EIS spectra using ZSimpWin software v. 3.60 (Ametek, EChem software, Ann Arbor, MI, USA) to analyze the parameters of each component of the overall impedance and their relationship with the electrolyte composition and the limiting anodization voltage.

## 3. Results and Discussion

### 3.1. Surface Appearance and Morphology

The formation of the PEO oxide coatings in this research proceeded via two-step method, whereby in the first step the galvanostatic treatment was applied to continuously growth the oxide layer up to the desired limiting voltage. At the moment of attaining the *U*_L_ the process was switched to the constant voltage mode and the current was allowed to drop to a few mA/cm^2^, which comprised the second step. The reason for applying of the second step was to stabilize the coating and to seal its weak spots. To see the current-voltage transients the reader is referred to our previous paper, where they were discussed in detail along with the effect of the processing parameters on the surface characteristics of the PEO coatings on tantalum [21]. The surface of the untreated Ta sample can be seen in Figure 1a,b. The scratches produced during grinding are visible to the naked eye, however, at the microscopic scale, the surface is rather smooth and essentially free from any distinguishing features.

During the PEO treatment of the tantalum specimens in all three solutions the process progressed through several stages. At first there was a sharp voltage increase in time, which was ascribed to the uniform thickening of the tantalum oxide passive layer (so-called conventional anodizing). In the second region of the galvanostatic treatment the voltage-time slope was observed to drop at the voltage of 150, 110 and 140 V for the solutions Ca1P2, Ca5P3 and (CaMg)5P3, respectively. This point in the treatment was corresponding to the breakdown of the oxide and the commencement of the PEO. The sparks were initially very small and moving fast across the treated surfaces, however, as the time went on they were starting to concentrate into more powerful discharges, which was termed as intensive sparking. This stage of the treatment commenced at approx. 300 V for the Ca1P2 solution and continued until it reached 400 V. The initiation of the intensive sparking during the PEO in the Ca5P3 solution was observed at the voltages as low as 170 V with the process still intensifying with time up to 400 V. In the case of the (CaMg)5P3 solution the intensive sparking commenced at approximately 200 V and progressed similarly as in the Ca5P3 solution up to the highest voltage studied here.

The morphology of the tantalum surface after the PEO processing was markedly altered by the presence of pores of varying size (Figure 1c–k). In general, the pore size increased with the *U*_L_ and with the concentration (and thus the conductivity) of the electrolyte. The samples obtained from the (CaMg)5P3 electrolyte were also found to have larger pores than then their counterpart coatings obtained in the Ca5P3 solution (Figure 1f–k). As it can be inferred from the insets in Figure 1, the tantalum specimens were uniformly coated with the oxide and their color depended on both the voltage and the composition of the electrolyte. Interestingly, the surface appearance was altered only slightly between the samples anodized up to 300 and 400 V. This was also reflected in the surface morphology, which changed drastically between 200 and 300 V for all samples, but the difference between those treated at *U*_L_ = 300 or 400 V was mainly in the pore size and their number. It is worth mentioning that the specimens prepared at *U*_L_ = 200 V resembled a rugged 2-D structure while the coatings obtained at higher voltages were characterized by the 3-D network of strut-like surface features.

### 3.2. Coatings Structure and Thickness

**Figure 2:**
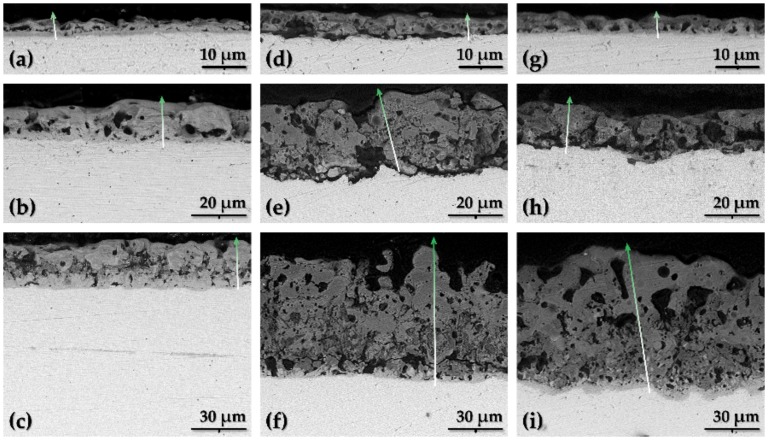
SEM cross-sectional images of the PEO oxide coatings on Ta substrate obtained from the solutions: Ca1P2 (**a**–**c**), Ca5P3 (**d**–**f**) and (CaMg)5P3 (**g**–**i**); and treated up to the limiting voltages: 200 V (**a**,**d**,**g**), 300 V (**b**,**e**,**h**) and 400 V (**c**,**f**,**i**); arrows show the paths of the EDX lines scans.

All the coatings could be divided into two sublayers: Outer porous layer and inner barrier layer at the metal/oxide interface. A third, intermediate sublayer could be discerned for some of the coatings, namely those on the samples treated at *U*_L_ = 400 V (Figure 2c,f,i). The outer layers are denser than the highly porous intermediate layers. This might be due to solidification and oxidation of the molten substrate ejected from underneath the forming porous oxide during the PEO [24,25,26]. This intermediate sublayer was especially thin for the Ca1P2 400 V sample (Figure 2c), when compared with those obtained from the Ca5P3 (Figure 2f) and (CaMg)5P3 (Figure 2i) electrolytes.

**Figure 3:**
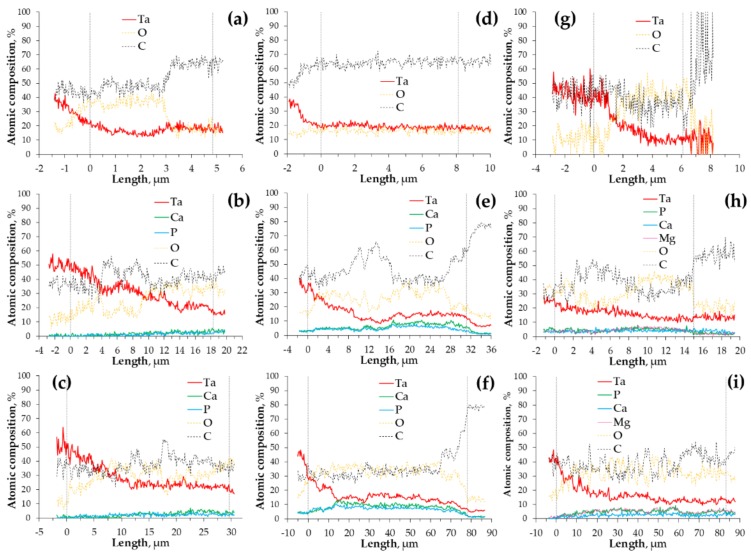
EDX line scans performed on the cross-sectional SEM images of Ca1P2 (**a**–**c**), Ca5P3 (**d**–**f**) and (CaMg)5P3 (**g**–**i**); and treated up to the limiting voltages: 200 V (**a**,**d**,**g**), 300 V (**b**,**e**,**h**) and 400 V (**c**,**f**,**i**); refer to Figure 2 to see the paths of the line scans.

To gain additional insight into the oxide films structure EDX line scans were performed on the samples along the paths depicted in Figure 2. The plots showing the variation of the atomic concentration as a function of the coatings’ depth are presented in Figure 3. None of the films obtained after the PEO up to 200 V were enriched with the electrolyte components in a way quantifiable by EDX (Figure 3a,d,g) as opposed to the coatings formed at higher voltages (Figure 3b,c,e,f,h,i). The reliability of EDX in terms of quantitation of light elements (i.e., carbon and oxygen) is questionable, however, their variation in atomic concentration across the coatings was included for the sake of comparison with the other components. The signals of carbon in the spectra originate from the resin, used for mounting of the Ta specimens for SEM observations. Some of the carbon might also be ascribed to the residue of the diamond paste from the polishing of the cross-sections. Nevertheless, the regions of the oxide films with relatively higher C content were identified as the regions of higher porosity since the pores could be filled with the resin during the mounting. The coatings obtained at the lowest voltage were found to be rather homogenous across their thickness. On the other hand, the films formed up to 300 V were observed to have significant porosity near the oxide-metal interface. For these samples, it was also noted that the concentration of the electrolyte species was higher in the outer parts of the coatings. The thickest of the oxide layers (formed up to 400 V) were the most porous (significant fluctuations in C content) and exhibited the accumulation of the Ca, P and Mg in the outer parts of the coatings (excluding the Ca5P3 400 V sample, for which it was found that most of the electrolyte components were present near the oxide-metal interface (Figure 3f), however, their concentration in the outer parts of the film was also relatively high).

**Figure 4:**
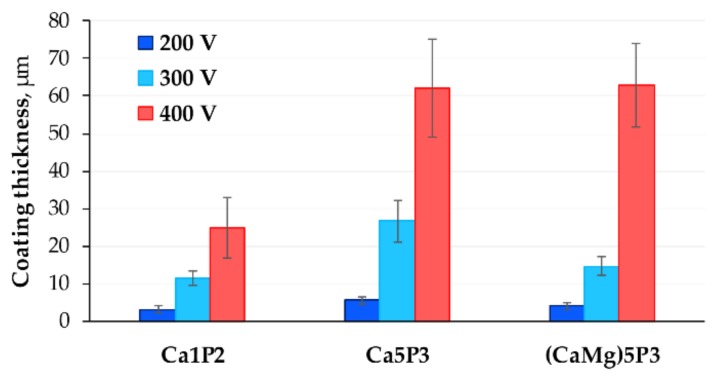
Average thicknesses of the PEO coatings formed on Ta.

The anodization voltage is one of the main parameters determining the total thickness of the oxide coating produced by the PEO [15]. Consequently, it is not surprising that the thickness of the coatings increased with the *U*_L_ and with the electrolyte concentration (Figure 4). The latter is responsible for exerting less resistance to the current flow during the treatment, which results in larger voltage drop across the forming oxide layer and thus intensifying the process [15,21]. Although it was recently suggested that the nature of the anions and their concentration influence the properties of the growing oxide and have a more definitive effect on the sparking regime and plasma formation mechanism than the conductivity of the electrolyte [27]. No linear relationship between the *U*_L_ and the total thickness of the coating could be established, probably because of the transition of the process through various sparking regimes as the voltage was increased. This resulted in nonlinear growth rate with respect to time, which is not encountered in the case of AC or pulsed bipolar variant of the PEO, where the process regime is controlled by the frequency, duty ratio and peak currents [28,29,30].

It is interesting to note that the addition of Ca^2+^ or Mg^2+^ ions had similar effect on the coating thickness when *U*_L_ = 400 V, however, the Ca5P3 200 V and Ca5P3 300 V samples were considerably thicker than the (CaMg)5P3 200 V and (CaMg)5P3 300 V. This might be because of the lower standard potential of calcium with respect to magnesium or the reason may stem from the counter ions used in their respective salts.

### 3.3. Electrochemical Impedance Spectroscopy

EIS helps to determine the total resistance of the biomaterial/electrolyte interface in the corrosive environment. It also enables one to break the total impedance down to components corresponding to different parts of the coating or surface processes [31].

**Figure 5:**
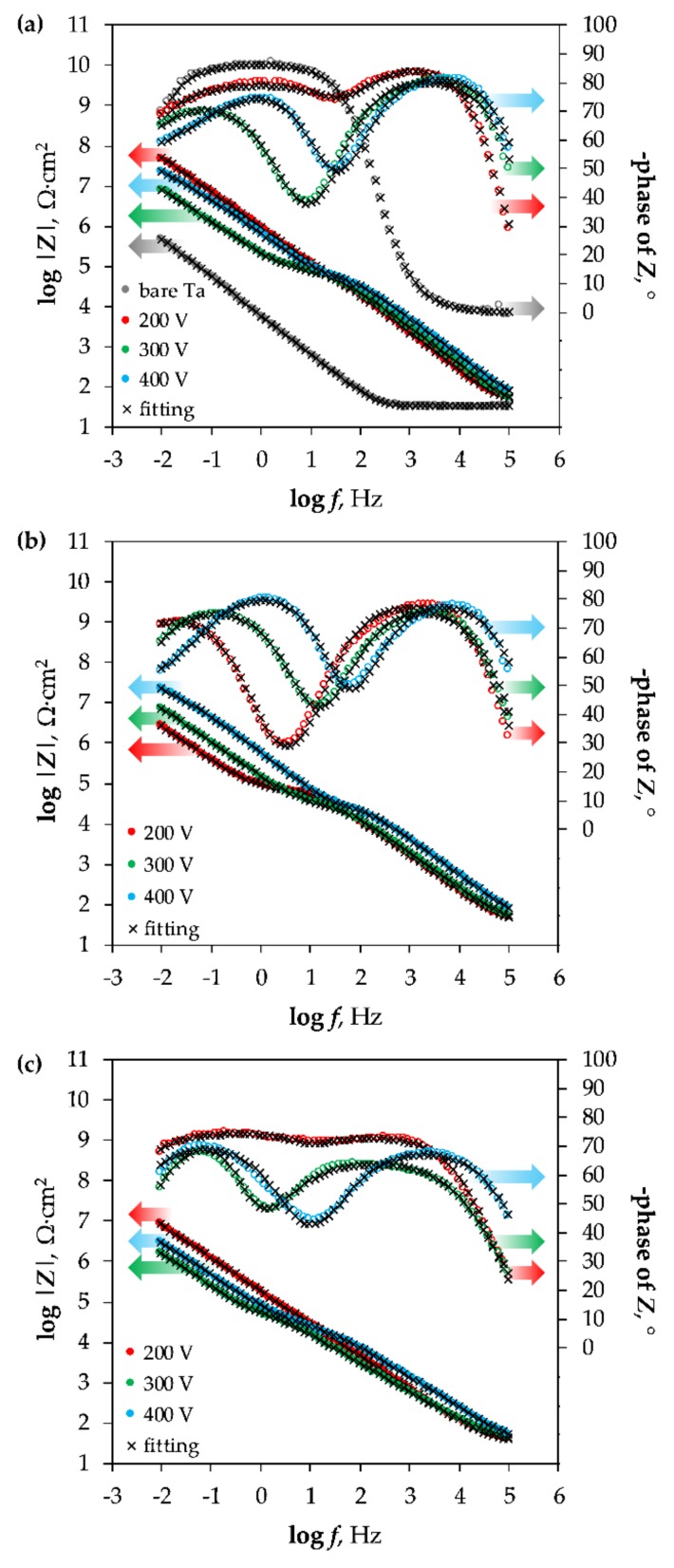
Bode plots of impedance spectra recorded for the Ta specimens before (bare Ta) and after the PEO in the solutions: Ca1P2 (**a**), Ca5P3 (**b**) and (CaMg)5P3 (**c**); arrows indicate which part of the plots correspond to the impedance magnitude points (**|*Z*|**), and the phase of *Z* values.

**Figure 6:**
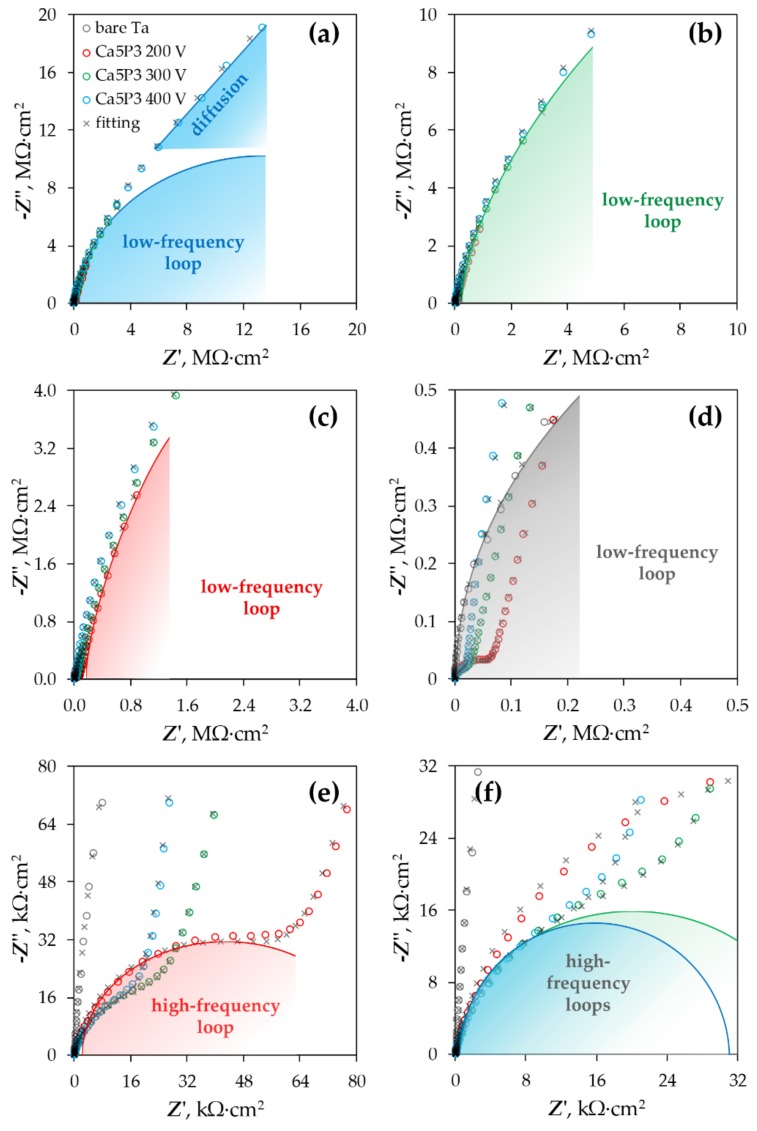
Nyquist complex plane plots showing the impedance results of the bare Ta sample and tantalum surfaces after PEO in the Ca5P3 solution conducted up to 200, 300 or 400 V; the same plots are shown in different magnifications to visualize the (**a**–**d**) low-frequency and (**e**–**f**) high-frequency capacitive loops.

The EIS spectra measured for the bare Ta substrate and the coated tantalum samples are presented as Bode plots in Figure 5, while the Nyquist complex plane plots can be observed in Figure 6. The latter are shown only for the Ca5P3 solution for the sake of visualizing of the capacitive loops discerned for the oxide films. The spectrum of the Untreated Ta is characterized by a single time constant, noticeable between the frequency of 10^0^ and 10^−1^ Hz (Figure 5a and Figure 6d), which can be associated with a parallel *RC* connection. For the tantalum surfaces after the PEO at least one extra time constant could be seen in the spectra (Figure 6). The impedance magnitude (|*Z*|) of the spectra measured for the PEO coatings on Ta was above the bare tantalum spectrum in the whole range of frequency. The second simplest impedance spectra can be ascribed to the Ta samples that were subjected to PEO at 200 and 300 V (Figure 5). Two well separated time constants can be observed in this case (Figure 6b,c,f). Slightly more complicated spectra were measured for the Ca1P2 400 V and Ca5P3 400 V samples (Figure 5a,b and Figure 6). This complication manifests itself as a close to linear region observed in the low-frequency end of the spectrum (Figure 6a). Such impedance responses might suggest that diffusion might play the crucial part in limiting the corrosion rate of Ta for these samples. Nevertheless, the impedance spectrum of the (CaMg)5P3 400 V sample lacks the linearity in the low-frequency region. This, combined with the relatively low impedance magnitude measured for this coating, might imply that the oxide film was more porous in this case and offered less diffusional resistance. The finding agrees with the cross-sectional investigations (Figure 2c,f,i and Figure 3c,f,i ) of the coatings. The highest impedance and consequently the highest corrosion resistance was observed for the PEO coating formed in the Ca1P2 solution at *U*_L_ = 200 V, while the one that provided the least resistance was prepared by the anodization in the (CaMg)5P3 solution at 300 V. Furthermore, a trend of the increasing of the coatings’ impedance with the *U*_L_ raised from 300 to 400 V was noted in each electrolyte. The coatings obtained at *U*_L_ = 200 V in Ca1P2 and (CaMg)5P3 solutions had similar surface morphology (Figure 1c,i), structure (Figure 2a,g and Figure 3a,g ), with the latter being slightly thicker (Figure 4). Furthermore, their EIS spectra were comparable. However, the coating obtained at the same voltage in the Ca5P3 solution resembled the one obtained at *U*_L_ = 300 V in the Ca1P2 electrolyte (Figure 1d,f; Figure 2b,d; Figure 4). This suggests that the sparking regime, necessary to obtain relatively thick (above 10 µm) coatings, was reached in the Ca5P3 solution below 200 V. This voltage was found to be higher in the other two electrolytes, however, below 300 V as it was described in our previous work on PEO of Ta [21].

**Figure 7:**
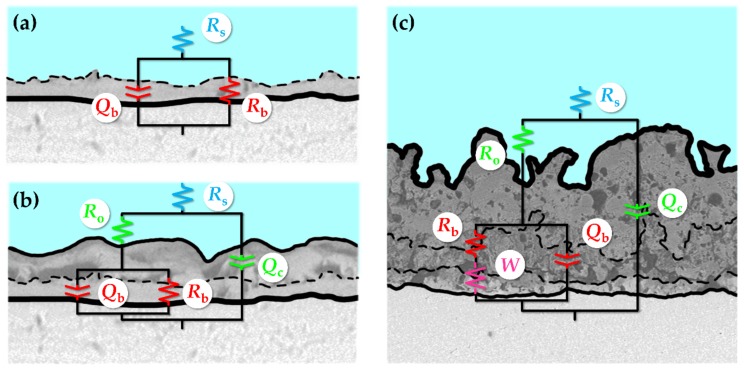
Equivalent electrical circuits used for fitting of the impedance spectra of the untreated Ta sample (**a**), the PEO samples that showed 2 time constants (**b**), and those that exhibited 2 time constants with a linear constant-phase region (**c**).

Knowing the number of time constants in the spectra it could be attempted to find suitable EECs to fit the data. The proposed circuits and the graphical representation of their physical meaning can be reviewed in Figure 7. 

As it is customary in the EIS analysis of the corroding systems, capacitors representing dielectric properties of the electrochemical interfaces were substituted by constant phase elements (CPEs or *Q*), to account for the non-ideality of the studied surfaces.

The impedance response of the CPE can be obtained from the following formula [32]:

(1) $$Z_{\mathrm{CPE}}=\frac{1}{Q{\left(j\omega \right)}^{n}}$$

where *n* parameter can attain values in the range <−1,1>. When *n* is close to +1 the CPE approximates the behavior of the ideal capacitor and *Q* has a meaning similar to that of capacitance. At *n* = 0 the *Z*_CPE_ reduces to the impedance of a resistor, while *Q* is numerically equal to the reciprocal of its resistance. The last of the extremes (*n* close to −1) signify situation when the CPE approximates the inductor with *Q* = 1/*L* (*L*—inductance). The deviation of *n* away from unity may correspond to the unevenness of the electrode surface, its chemical inhomogeneity and the variable oxygenation of the electrolyte with depth [32]. A special case of the CPE was also included in the circuit modeling the impedance response of the Ca1P2 400 V and Ca5P3 400 V samples—Warburg element (*W*). For *W* the value of *n* equals to 0.5 because this element is used to model the semi-infinite linear diffusion, which is a function of the square-root of time. The impedance response of Warburg can also be written in the form of separated real and imaginary contributions:

(2) $$Z_{W}=\frac{\sigma }{\omega^{0.5}}-j\frac{\sigma }{\omega^{0.5}}$$

where *σ* is the Warburg coefficient. By combining Equation (1) with Equation (2) one can arrive with the relationship between the *Q* parameter of Warburg element and the *σ*:

(3) $$\sigma =\frac{1}{Q_{W}\sqrt{2}}$$

The corrosion behavior of the untreated Ta surface was modeled with the use of a simple *R*(*QR*) circuit (Figure 7a). *R*_s_ is the resistance of the electrolyte between the tip of the reference electrode capillary and the investigated sample and does not give any insight into its corrosion resistance. However, it must be included in the overall model. The same circuit was reported by others who studied the corrosion of tantalum either in its pure form or as a coating [2,8,33,34]. The *R*_b_ and *Q*_b_ connected in parallel represent the resistance and capacitance of the passive barrier layer forming on the submerged Ta surface. 

There is much controversy in the literature where it comes to the appropriate EEC, which would accurately approximate the behavior for the corroding PEO coating and at the same time provide a suitably good fit of the experimental data to the model. Most of the time, the researchers are utilizing the simplest possible EEC to perform the fitting procedure—*R*(*Q*[*R*(*QR*)]) [35,36,37,38]. The inclusion of the Warburg impedance into the second nested (*QR*) pair is sometimes practiced [39], nevertheless, sometimes the values of this parameter were observed to be unreasonably high. The addition of an extra time constant in the form of another nested (*QR*) pair yielding *R*(*Q*[*R*(*Q*[*R*(*QR*)])]) or *R*(*QR*)(*QR*)(*QR*) could also be found in the literature [40,41,42,43]. This, however, requires the presence of a tri-layered structure to be reasonable. Recently, Alves et al. [44] proposed an interesting circuit (*R*(*Q*[*RQ*][*R*(*QR*)])), which accounted for the appearance of the third time constant in the corroding PEO coating EIS spectrum.

It is known that the inclusion of extra parameters in the circuit model increases the number of degrees of freedom of the system, and therefore, provides a better fit to the experimental data. However, it must be remembered that the circuit parameters require a physical explanation to give meaningful information of the interface under investigation. Consequently, relatively simple equivalent circuits were used in the research. All spectra of the PEO coatings were fitted to the EECs having two time constants (Figure 7b) with the exception of the Ca1P2 400 V and Ca5P3 400 V samples, for which the circuits were additionally modified with Warburg impedance (Figure 7c). The *R*_o_ and *Q*_c_ pair represents the extra time constant not present in the model from Figure 7a. *R*_o_ is the resistance of the electrolyte in the pores of the outer porous oxide layer, while *Q*_c_ stands for the total capacitance of the PEO coating. *W* represents in the circuit from Figure 7c is the Warburg impedance, which models the diffusional contribution to the total impedance of the PEO coatings.

Circuit parameters obtained from the fitting of the EIS spectra to the EECs from Figure 7 are shown in Table 1. Chi-squared values obtained from the fitting of the results were below 4.05 × 10^−3^. As encountered in many instances in the literature [35,37,42,43,45,46], the main factor that determines the overall corrosion resistance of the metals and alloys surfaces modified by PEO was found to be the resistance of the barrier sublayer. The same relationship regarding the corrosion resistance of Ta as this found in Figure 5 was reflected in the calculated results.

The barrier layer resistance (*R*_b_) of the Untreated Ta sample was much lower than that published by Hee et al. [33], who found it to be one order of magnitude higher (12.4 MΩ·cm^2^). However, the bare tantalum surface in the cited research spent 14 days in an SBF and was not etched beforehand, which means that it had much more time to passivate and homogenize the barrier oxide layer. The results of corrosion resistance investigations of nanostructured Ta coating and annealed Ta reported by Fattah-Alhosseini et al. [8] conform to that idea. In their work, the researchers found that the *R*_b_ increased to 0.947 and 3.71 MΩ·cm^2^ after 6 h of immersion, for the coating and annealed Ta, respectively. After 24 h the resistance was still increasing, suggesting that Ta was in the passive state. In fact, in the article published by the same group [34], the evolution of the corrosion circuit parameters of commercially pure Ta in Ringer’s solution was investigated. The results show that after 6 h of immersion in the electrolyte the *R*_b_ was equal to 1.22 MΩ·cm^2^, which is only slightly less than the value obtained in the present study. From the parameters calculated for the PEO oxide films (Table 1), it can be seen that the values of the *n* parameters in all of the cases were higher than 0.75, which indicates that they are approximating the capacitance of the different portions of the coatings (namely, the porous and the barrier sublayers). As previously mentioned, this parameter is connected with the uniformity of the oxide layers in terms of their structure and chemical composition. No appreciable changes in the *n* parameters were found in the coatings prepared in the Ca1P2 and Ca5P3 solutions with the increasing limiting anodization voltage. However, for the (CaMg)5P3 solution it was found that *n* was decreasing with the increasing *U*_L_. The effect was especially pronounced for the (CaMg)5P3 400 V sample. This is observed because of the highest porosity of the coating among the all studied PEO films. 

The values of *Q*_c_ and *n*_c_ correspond to the capacitance of the entire coating, which is an important corrosion resistance parameter, and can be approximated from each of the following expression [8,32,34,44,47]:

(4) $$C_{\mathrm{eff},1}=\frac{\sqrt[{n}]{QR}}{R}$$

where *R* and *Q* are the parameters belonging to a pair of circuit elements connected electrically in parallel. The calculated effective capacitances (*C*_eff_) can be seen in Table 2.

The capacitance of a capacitor is given by the relationship [8,34,44]:

(5) $$C=\frac{\varepsilon \varepsilon_{o}A}{\delta }$$

where *ε* stands for the relative permittivity (or dielectric constant) of the dielectric material forming the capacitor, *ε*_o_ is the permittivity of vacuum, *A* is the active surface area and *δ* corresponds to the dielectric’s thickness. Therefore, the capacitance is providing one with the information regarding the total thickness of the coating and its penetration with the electrolyte, having significantly higher dielectric constant than tantalum oxide and other phases that were present in the PEO films [21]. Knowing the thicknesses from Figure 4, it can be reasoned, which of the coatings had higher porosities and were more likely to be penetrated by the electrolyte during the measurements.

For the Ca1P2 and Ca5P3 solutions, the capacitances were found to be decreasing as the *U*_L_ was increasing. This is reasonable, as the coating thicknesses also increased with the limiting anodization voltage (Figure 4). However, the Ca1P2 coatings were considerably thinner than their Ca5P3 counterparts, meaning that the electrolyte penetration through the internal porosity of the latter oxide films was observed to a higher extent. From Figure 5 and Table 1 it can be seen that for the coatings obtained from the (CaMg)5P3 solutions there was no need to include the Warburg impedance into the EEC (Figure 7b,c) to arrive with the reasonably good fit of the EIS data, even for the (CaMg)5P3 400 V sample. However, from Figure 2f,i and Figure 4, it is evident that it is of comparable thickness with the Ca5P3 400 V film. This implies that the coating was fully wetted by the Ringer’s solution at the time of the measurement, which was also evident from the high capacitances in Table 2. Indeed, the capacitance of the (CaMg)5P3 300 V was much higher than that PEO-ed at *U*_L_ = 200 V and 400 V. The latter two had approximately equal values of the *C*_eff_, although the thickness of the (CaMg)5P3 400 V sample was an order of magnitude higher (Figure 4). Therefore, it can be stated that the coatings obtained in the (CaMg)5P3 solution were the easiest to penetrate by the corrosion liquid, compared to the other studied solutions. This also may suggest that the Warburg impedance in the Ca1P2 400 V and the Ca5P3 400 V samples’ EIS spectra might have arisen from the fact that there was a portion of the coating still not penetrated by the electrolyte. This hypothesis will be tested in our future work where the long-term corrosion studies of the coatings will be performed.

### 3.4. Polarization Experiments

**Figure 8:**
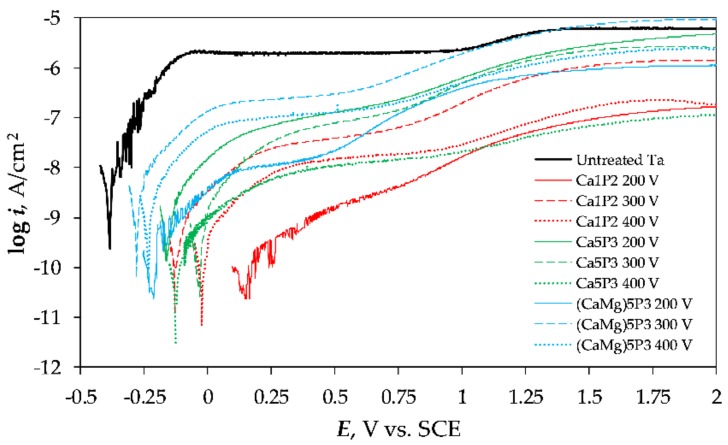
Potentiodynamic polarization curves measured in Ringer physiological solution for the Ta samples before (Untreated Ta) and after the PEO.

The tightness of the coatings and their resistance to the breakdown was tested by subjecting them to polarization scans up to +2 V vs. SCE (Figure 8). The points around the corrosion potential (*E*_cor_ or *E*_OC_) were obtained from the potentiostatic measurements to minimize the effect of dielectric charging of the coatings. The PDP curve of the Untreated Ta surface was also included in Figure 8 as a background. It can be immediately seen that the currents registered for the PEO-treated Ta specimens were almost always lower than the ones belonging to the bare tantalum save from the (CaMg)5P3 300 V sample. The current measured for the latter coating exceeded the passive current of the Untreated Ta at ca. 1.25 V vs. SCE. The currents measured in the electrochemical experiments were normalized by the active surface area of the samples. However, the geometrical area was much smaller than the real area for any surface, which was not completely flat. Therefore, although the coating existed on the tantalum surface, it provided more surface area for the current to flow, which also impacted the corrosion resistance negatively. No evidence of pitting corrosion or oxide breakdown was discovered from the measured data.

The corrosion parameters, such as *E*_cor_, *R*_p_ (polarization resistance) were extracted from the DC experiments and can be seen in Table 3. 

The values of the *R*_p,LPR_ were obtained by taking the slope of the *E* vs. *i* relationship in the range ±10 mV vs. *E*_cor_. The *R*_p_ was also calculated from the EIS fitting results according to the formulae [48]:

(6) $$R_{p,\mathrm{EIS}}=R_{s}+R_{b}$$

(7) $$R_{p,\mathrm{EIS}}=R_{s}+R_{o}+R_{b}$$

(8) $$R_{p,\mathrm{EIS}}=R_{s}+R_{o}+R_{b}+\frac{\sigma }{\sqrt{\omega }}$$

using the values from Table 1. The maximum current density measured for each studied surface was termed as *i*_peak_ and it corresponds to the tightness of the PEO coating.

The formation of coatings not only shifted the corrosion potential towards more positive values (Figure 8, Table 3) but also resulted in higher polarization resistance. The shift was higher for the more resistive coatings. In nearly all the cases the PEO treatment resulted in the increase of *R*_p_ by one or two orders of magnitude. It is worth noting that the impedance results agree with those extracted from the polarization measurements, however, the values obtained from EIS were in most of the case smaller than those of the DC experiments (Table 3). By comparing the *i*_peak_ values it can be seen that while the *R*_p_ increased the maximum measured currents were smaller. However, the decrease was not proportional to the increase of the *R*_p_ between the samples. This can be ascribed to the partial dissolution of the coatings during the scan, which is to be expected of the coatings that are aimed to increase the bioactivity by providing the bio-elements at the implantation site.

## 4. Conclusions

This work described the corrosion resistance of the DC PEO coatings prepared on commercially pure tantalum. The experiments were carried out in Ringer’s solution at 37 °C. The structure and impedance response of the coatings was determined by EIS and their tightness was measured by PDP. The corrosion resistance of Ta after the PEO was found to be the best for the surfaces that were treated at the voltages below the onset of intensive sparking. This was found for the Ca1P2 200 V and (CaMg)5P3 200 V samples. However, for the samples anodized in the Ca5P3 solution, it was found that the *U*_L_ = 200 V was high enough to observe the effects of this PEO regime. After the onset of intensive sparking it was noted that the oxidation conducted up to higher voltages (i.e., 400 V) led to much better results compared to the coatings formed at the intermediate voltage (300 V). This was owed to the higher resistance of the barrier layer, even for the more porous coatings produced at 400 V or to the effects associated with the diffusion in the pores. In our future work the long-term EIS investigations, aimed at verifying whether the Warburg impedance will lose its significance as the electrolyte penetrates the coatings over time, will be performed.

## Figures and Tables

**Table 1:** Circuit parameters calculated from the fitting of the EIS spectra to the EECs from Figure 7.

Solution	-	Ca1P2			Ca5P3			(CaMg)5P3		
*U*_L_, V	Untreated	200	300	400	200	300	400	200	300	400
*R*_s_, Ω·cm^2^	32.0 ± 1.3	35.4 ± 6.1	27.7 ± 2.2	34.1 ± 1.0	32.4 ± 1.4	31.2 ± 1.8	34.0 ± 2.0	30.3 ± 5.7	34.6 ± 4.2	27.8 ± 5.9
*Q*_c_, s*^n^*/(GΩ·cm^2^)	-	143 ± 55	102 ± 16	60.0 ± 1.4	226 ± 68	178 ± 80	83.4 ± 10.5	471 ± 65	3060 ± 370	538 ± 207
*n*_c_	-	0.94 ± 0.02	0.92 ± 0.01	0.93 ± 0.00	0.89 ± 0.02	0.89 ± 0.03	0.90 ± 0.01	0.86 ± 0.01	0.75 ± 0.02	0.79 ± 0.02
*R*_o_, kΩ·cm^2^	-	57.0 ± 20.4	89.3 ± 8.8	64.3 ± 6.4	70.8 ± 3.8	38.4 ± 6.0	31.4 ± 3.4	60.8 ± 28.5	90.4 ± 16.9	31.7 ± 1.6
*Q*_b_, s*^n^*/(GΩ·cm^2^)	24,600 ± 3200	138 ± 38	1160 ± 170	243 ± 24	4190 ± 1270	1050 ± 280	228 ± 38	39.8 ± 9.0	3970 ± 1670	2030 ± 450
*n*_b_	0.97 ± 0.00	0.82 ± 0.01	0.83 ± 0.01	0.90 ± 0.02	0.84 ± 0.01	0.84 ± 0.03	0.89 ± 0.06	1.00 ± 0.00	0.95 ± 0.04	0.78 ± 0.03
*R*_b_, MΩ·cm^2^	1.67 ± 0.22	186 ± 88	41.0 ± 10.5	5.04 ± 3.98	14.1 ± 4.0	32.9 ± 5.3	18.1 ± 5.2	82.7 ± 14.9	5.34 ± 2.17	25.4 ± 7.3
*σ*, MΩ·cm^2^/s^0.5^	-	-	-	5.75 ± 0.93	-	-	5.91 ± 2.09	-	-	-
*χ*^2^ × 10^4^	<8.81	<5.16	<14.5	<8.78	<40.5	<11.2	<11.7	<4.17	<6.42	<15.6

**Table 2:** Effective capacitances of the PEO coatings on tantalum.

Solution	*U*_L_, V	*C*_eff,1_, nF/cm^2^	*C*_eff,2_, nF/cm^2^
Ca1P2	200	177 ± 83	156 ± 66
	300	116 ± 20	98.0 ± 15.1
	400	66.5 ± 5.0	57.7 ± 4.9
Ca5P3	200	262 ± 97	208 ± 70
	300	231 ± 125	180 ± 89
	400	103 ± 16	85.9 ± 12.2
(CaMg)5P3	200	864 ± 190	639 ± 131
	300	7570 ± 600	4110 ± 730
	400	1070 ± 540	658 ± 296

**Table 3:** Corrosion parameters acquired from the electrochemical measurements.

Solution	*U*_L_, V	*E*_cor_, mV vs. SCE	*R*_p,LPR_, MΩ·cm^2^	*R*_p,EIS_, MΩ·cm^2^	*i*_peak_, µA/cm^2^
Untreated	0	−371.1 ± 58.5	3.52 ± 0.41	1.67 ± 0.22	5.91 ± 3.67
Ca1P2	200	142.7 ± 97.7	593 ± 143	186 ± 88	0.341 ± 0.257
	300	−102.3 ± 52.8	53.1 ± 17.2	41.1 ± 10.5	1.59 ± 0.18
	400	−69.9 ± 41.0	137 ± 48	63.4 ± 13.4^*^	0.169 ± 0.069
Ca5P3	200	−166.6 ±18.5	26.2 ± 17.6	14.2 ± 4.0	4.17 ± 0.56
	300	−81.2 ± 53.8	37.5 ± 7.4	33.0 ± 5.8	2.04 ± 1.19
	400	−78.7 ± 89.4	216 ± 153	84.2 ± 33.2^*^	0.155 ± 0.129
(CaMg)5P3	200	−184.2 ± 45.4	225 ± 73	83.7 ± 10.7	1.96 ± 1.69
	300	−307.2 ± 56.6	7.04 ± 1.28	5.43 ± 2.16	7.15 ± 5.03
	400	−202.6 ± 48.7	15.6 ± 4.4	25.4 ± 7.3	3.24 ± 0.76

* The diffusional resistance was calculated as the real part of the Warburg impedance at the frequency of 10 mHz.
